# Damage-evoked signals in cochlear neurons and supporting cells

**DOI:** 10.3389/fneur.2024.1361747

**Published:** 2024-02-14

**Authors:** Megan Beers Wood, Nate Nowak, Paul Albert Fuchs

**Affiliations:** ^1^The Center for Hearing and Balance, Otolaryngology-Head and Neck Surgery, Baltimore, MD, United States; ^2^The Department of Neuroscience, Johns Hopkins University School of Medicine, Baltimore, MD, United States

**Keywords:** cochlea, type II afferent, epithelia, trauma, calcium waves, hyperacusis

## Abstract

In addition to hearing loss, damage to the cochlea can lead to gain of function pathologies such as hyperacusis. It has been proposed that painful hyperacusis, noxacusis, may be carried to the central nervous system by type II cochlear afferents, sparse, unmyelinated neurons that share morphological and neurochemical traits with nociceptive C-fibers of the somatic nervous system. Also like in skin, damage elicits spreading calcium waves within cochlear epithelia. These are mediated by extracellular ATP combined with IP3-driven release from intracellular calcium stores. Type II afferents are excited by ATP released from damaged epithelia. Thus, the genesis and propagation of epithelial calcium waves is central to cochlear pathology, and presumably hyperacusis. Damage-evoked signals in type II afferents and epithelial cells have been recorded in cochlear explants or semi-intact otic capsules. These efforts have included intracellular electrical recording, use of fluorescent calcium indicators, and visualization of an activity-dependent, intrinsic fluorescent signal. Of relevance to hyperacusis, prior noise-induced hearing loss leads to the generation of prolonged and repetitive activity in type II neurons and surrounding epithelia.

## 1 Introduction

The mammalian cochlea has a problem. The evolution of sensitive hearing over a wide frequency range has produced a biomechanically complex sense organ, but one vulnerable to irreparable damage. Adding insult to injury, the associated pathology of hyperacusis can undermine quality of life as much or even more than partial hearing loss *per se*. Thus, efforts continue to identify the underlying pathological mechanisms of hyperacusis. A single row of inner hair cells and their associated type I afferents encode and transmit acoustic information to the brain. Three rows of outer hair cells are inhibited by cholinergic efferent neurons. A much smaller number (5% of the total) of unmyelinated type II afferents extend hundreds of microns along the cochlear length to contact dozens of outer hair cells. Previous work has examined the hypothesis that type II cochlear afferents may be not only acoustic sensors (1–3), but additionally respond to tissue damage as potential inner ear nociceptors (4–6). Their role in activation of the medial olivocochlear (MOC) neurons remains to be delineated fully (7–9), although type II afferents are unlikely to provide the dynamic range and frequency selectivity of the MOC efferents (10, 11). Of equal importance, it is known that local damage in cochlear explants can trigger intercellular calcium signals that propagate through surrounding epithelia (12, 13). These calcium waves may share features with the epithelial waves (14, 15) that modulate hair cell driven afferent activity (16–18) prior to the onset of hearing (19). This mini-review describes the propagation of damage-evoked epithelial calcium waves and relates them to the activity of type II cochlear afferents.

## 2 Calcium waves

Experiments on cochlear damage can be informed by the extensive studies of neural and epithelial mechanisms of somatic pain (20). Relevant to this review are those that have examined the propagation of damage-evoked calcium waves in skin epithelia (21, 22) that modulate or activate pain-sensing C-fibers (20). A particularly informative *in vivo* study was carried out on earlobe skin of mice expressing a genetically-encoded calcium indicator (23). Calcium waves triggered by laser ablation of single keratinocytes were recorded, subjected to pharmacological blockade and used to inform a model of propagation. This model combined ATP release through connexin hemi-channels with IP3-evoked release of calcium from internal stores. The model accurately described experimentally-observed calcium waves whose velocity fell with time and distance from the site of lesion, with initial speeds averaging ~25 μm/s, then falling to zero within ~13 s elapsed time, or 70 μm traveled. Calcium imaging also has been used to characterize damage-evoked epithelial waves in cochlear explants (12, 13, 24, 25). As in skin, these intercellular calcium waves depend on released ATP as an extracellular signal, and IP3-dependent release of calcium from cytoplasmic stores (26). Similarly to those in living skin, *ex vivo* cochlear calcium waves slow from an initial velocity of 10–15 μm/s with distance from the site of lesion (13, 24). These commonalities suggest that cochlear epithelial waves may activate type II afferent neurons to drive painful percepts, by analogy to the role of C-fibers in skin. Thus it would be informative to record damage-evoked activity simultaneously in both epithelial and type II afferents in cochlear explants.

## 3 Activation of type II cochlear afferents

Type II cochlear afferents have small caliber, unmyelinated processes that extend hundreds of microns toward the cochlear base, contacting dozens of outer hair cells enroute (27–29). Type IIs make up only a small fraction (~5%) of cochlear afferents, the great majority being myelinated type I afferents postsynaptic to inner hair cells (30, 31). Given the scarcity and small size of type II afferents, it is not surprising that only few extracellular recordings have been obtained *in vivo*, showing limited acoustic sensitivity (2, 3); although c-FOS labeling of brainstem targets of type II afferents did find activity induced by loud, non-damaging, sound (1). Whatever the acoustic sensitivity of type II afferents, they must be far less able to support discriminative hearing than type I afferents whose activity accounts for acoustic intensity, frequency and timing (32). Intracellular tight-seal recordings from type II afferents in cochlear explants revealed that glutamatergic transmission from one OHC was orders of magnitude weaker than that provided by each IHC to type I afferent ribbon synapse (4, 33, 34). But in addition, type II afferents are strongly depolarized by direct application of ATP (4), a known algogenic mediator in skin (35). Acute mechanical damage to OHCs (produced by a glass probe) caused a large, long-lasting depolarization of the type II afferent that could trigger bursts of action potentials (5). The underlying inward current was ATP and connexin dependent and resulted from the activation of both P2X and P2Y purinergic receptors. The proposed hypothesis was that ATP released from surrounding supporting cells activated the type II afferent. Testing this hypothesis further requires a tissue-wide method for recording type II activity from mature animals.

## 4 Calcium imaging of type II afferents

Dissection damage and tissue degradation make intracellular recording from type II afferents limited largely to explants from the apical turn of early postnatal cochleas. Thus, subsequent studies have turned to fluorescence imaging in semi-intact cochlear capsules (‘half-shells’) (24). Using multi-photon microscopy, it is possible to interrogate any region of cochleas removed from adult mice (36). Several transgenic mouse lines have been developed to provide expression of the calcium indicator GCaMP6f in type II cochlear afferents (37–39). Calcium indicators can be expressed in apical type II afferents employing a tyrosine hydroxylase promoter (39). The dopamine type 2 receptor promoter functions preferentially in basal type II afferents (38). A third line utilizing the Tac1 promoter drives expression in type II afferents throughout the cochlea, combined with a tonotopic gradient of expression in epithelial cells (36). For the velocity measurements described below, the floxed GCaMP6f mouse (C57BL/6N-Gt(ROSA)26Sortm1(CAG-GCaMP6f)Khakh/J IMSR catalog #JAX:029626, RRID:IMSR_JAX:029626) was crossed with a tyrosine hydroxylase promoter Cre-recombinase mouse, Th^2A^CreEr (40). Following laser ablation of one to three OHCs, type II afferents had increased GCAMP6f fluorescence that decayed over 40–50 s, similar to the time course of damage-evoked inward current observed in tight-seal, whole-cell recordings (5). This time course also is similar to that of calcium signals evoked in neonatal cochlear and vestibular tissue by pulsed application of ACh or ATP (41). The ATP receptor antagonist PPADs reduced the spatial spread of the type II afferent GCaMP6f response, accounting for the reduced duration of ATP-evoked current observed in voltage clamp of type II afferents (5) (i.e., less delayed excitation from distant regions). These observations are consistent with an hypothesis whereby ATP released during damage-evoked epithelial calcium waves drives the type II afferent response.

How does the type II afferent response relate to previously described epithelial calcium waves? It is not possible to answer this question by GCaMP expression alone, since epithelial cell signals would obscure the type II neuron’s response (but see Tac1 below). Fortunately, there is a much weaker but still visible damage-evoked fluorescence signal in epithelia not expressing GCaMP (as confirmed by negative immunolabeling, and presence in mice without GCaMP expression) that could serve as an indicator of epithelial damage. The possible genesis of Non-GCaMP Associated Fluorescence (NGAF) will be discussed below (36). The validity of NGAF measures was supported by the Tac1 mouse model that drives GCaMP expression in most, if not all type II neurons, but in a declining gradient of epithelial cells from apex to base, providing direct comparison of GCaMP fluorescence from type II afferents and NGAF in epithelial cells stochastically-expressing GCaMP in middle cochlear segments. These showed similar durations for the neuronal/epithelial GCaMP responses and NGAF. The occurrence of NGAF and neuronal responses was strongly correlated across all mouse models and experimental conditions (36). Moreover, PPADS reduced the integrated magnitude of the NGAF response as well as the spatial spread of the type II afferent GCaMP6f response (36). Thus, the NGAF signal was used as a proxy for damage-evoked epithelial waves described previously using calcium indicators (12, 13, 24, 25), and in particular whether these activities were altered by noise-induced hearing loss.

### 4.1 The effect of prior noise damage

Everyone has type II afferents, but not everyone has hyperacusis. Hyperacusis can be associated with a number of factors ranging from traumatic brain injury to autoimmune disease. Most commonly, it is related to prior acoustic trauma.^1^ Thus, any pathogenic mechanism should reflect this sensitization by previous trauma, akin to allodynia in skin. To that end, the cochlear damage response was examined in adult mice suffering noise-induced hearing loss (NIHL) (36). A striking feature of these NIHL cochleas was the occurrence of substantially delayed signals in both type II afferents and epithelial cells (NGAF). In both neurons and epithelia these could be repetitive, on the order of 1–2 per minute. These delayed damage responses were absent from immature (pre-hearing) cochlear tissue, and four times more likely in noise-exposed, compared to control adult tissue. Taken together these results support a hypothesis whereby tissue damage or stress activates ATP release from cochlear epithelia that can activate type II afferent neurons. Furthermore, prior trauma sensitizes the tissue leading to longer-lasting activity.

### 4.2 NGAF wave velocity

**Figure 1:**
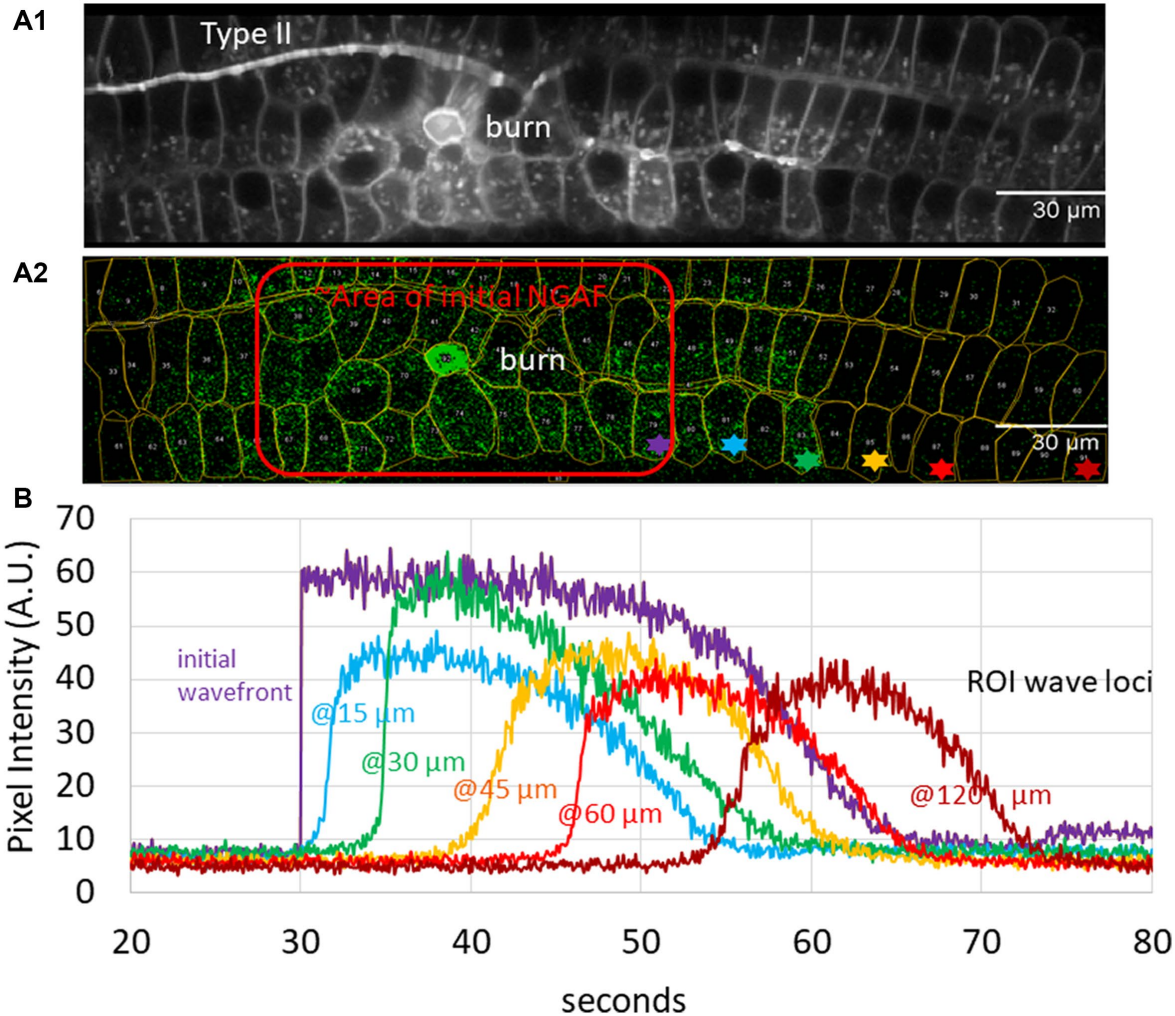
Analysis of autofluorescence – (Non-GCaMP Associated Fluorescence [NGAF]) in damaged cochlear epithelium; images from Th^2A^CreEr x Gcamp6f^fl/fl^ mouse tissue of Nowak et al. (36). **(A1)** Standard deviation image of time series z-projection spanning 5.5 min in total (80 ms/slice). A central region of initial damage (“burn”), and a type II neuron are visible. **(A2)** A single 80 ms slice 14 s after the focused laser ablation (central bright green circle) shows one time point of spreading NGAF fluorescence. Red outline shows approximate initial response area (burn plus 9 s). Regions of interest (ROIs) from A1 overlaid. Colored asterisks denote exemplar ROIs used to determine wave velocity. **(B)** Average pixel intensity from Z projection for asterisked ROIs in A2.

An unresolved issue concerns the use of NGAF for comparison to calcium waves. While there are a number of correspondences to support that assumption, further quantitative analysis is helpful. The velocity of calcium waves measured in both skin and cochlear tissue (see section 1, “calcium waves”) provides a useful measure for comparison to NGAF waves in the cochlea. Wave velocity following laser ablation was measured by first visualizing a standard deviation image of the entire time sequence to establish regions of interest (ROIs) for local measurement (Figure 1A1).

**Figure 2:**
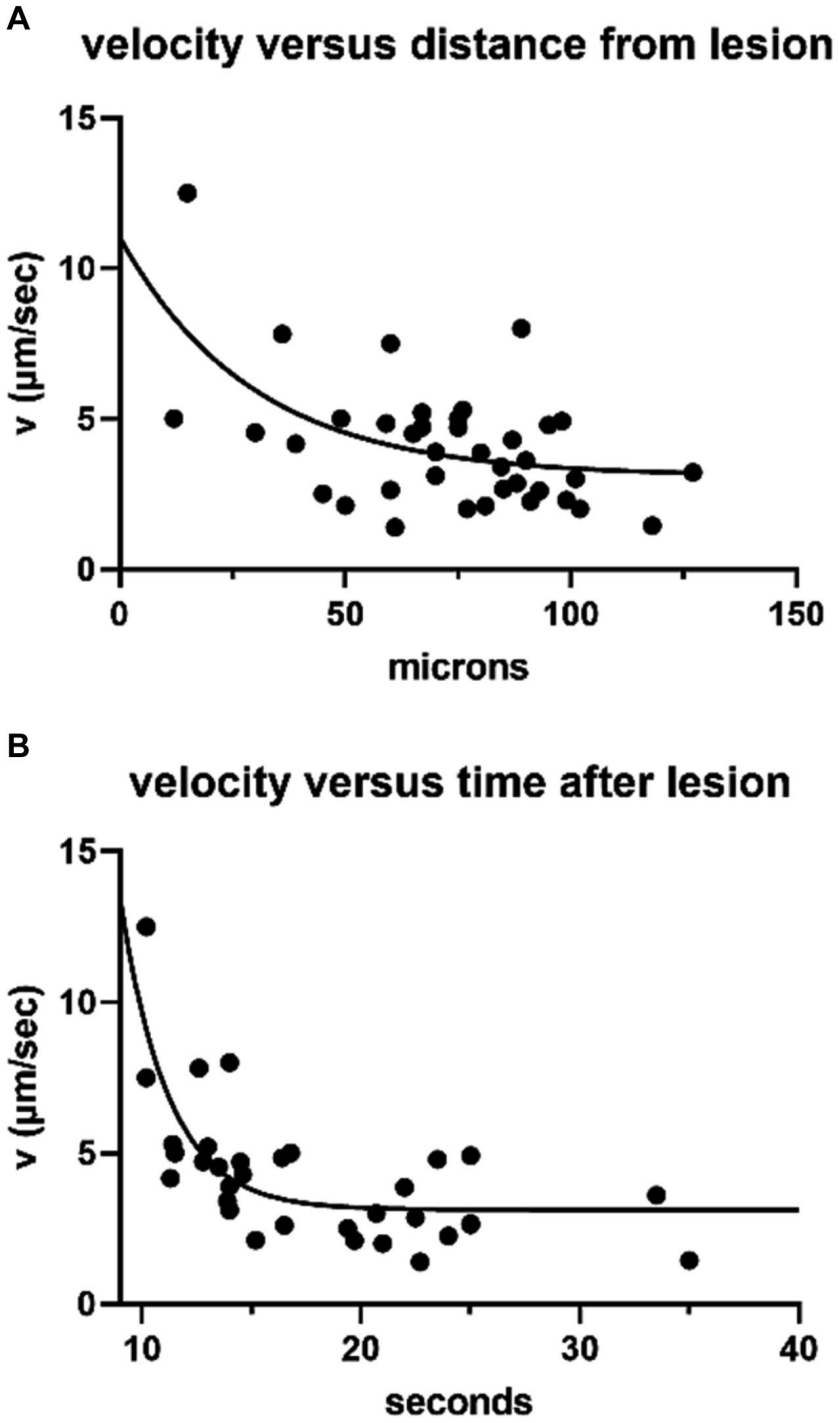
NGAF wave speed (38 ROIs, 10 trials on 6 cochleas) obtained as in Figure 1. **(A)** Wave speed with distance from leading edge of the initial response area (Spearman’s correlation = −0.39, *p* = 0.014), single exponential fit. **(B)** Wave speed with time (same ROIs as in **A**) >9 s after lesion, single exponential fit (Spearman’s correlation = −0.56, *p* = 0.003).

Average pixel intensity throughout the time series was recorded for exemplar ROIs then displayed as a function of distance and time from the initial lesion (Figure 1B). The change in distance with time between wave fronts (measured at the transition to the steepest part of the rising signal in each ROI, approximately 10% of the peak amplitude) was used to calculate the velocity as a function of distance and time (Figure 2). Initial wave speed could reach 13 μm/s but then fell to 2–4 μm/s with distance and time from the initial response to damage. Wave speed was negatively correlated with both distance (Spearman’s *r* = −0.39) and time (Spearman’s *r* = −0.56). Camera saturation prevented imaging for the first 9 s after laser ablation so the very first (fastest) waves were not captured, weakening the correlation. (The first NGAF images 9 s after damage were large, averaging 2,647 ± 1,302 SD μm^2^ in area. Enclosed by a red line in Figure 1A2) The overall pattern is like that of damage-evoked calcium waves in skin *in vivo* that fall from a high of 25 to 0 μm/s over 13 s (23), or the slow waves in *ex vivo* cochlear Deiters cells that slow from ~12 to 3 μm/s over ~100 μm (24). Thus, allowing for experimental differences, NGAF waves behave similarly to damage-evoked calcium waves in skin and cochlea, presumably by similar mechanisms involving extracellular ATP and IP3-dependent release from internal calcium stores.

But what is NGAF, if not ‘GAF’? What other activity-dependent fluorescence changes in the cells could be calcium associated? One possibility is the intrinsic fluorescence of the reduced form of the redox cofactor, nicotinamide adenine dinucleotide, NADH, that absorbs at 340 ± 30 nm and emits at 460 ± 50 nm wavelength (42). This spectral characteristic is lost when oxidized to NAD. This change in fluorescence has made NAD/NADH fluorescence a useful indicator of the redox state and so the metabolism of living cells (43). Of relevance here, a rise in cytoplasmic calcium enters mitochondria via the calcium uniporter to increase NADH production (44) and so fluorescence. Indeed, NADH fluorescence, cytoplasmic and mitochondrial calcium are tightly correlated in other tissues (44) and can rise and fall with a half cycle duration of ~10 s (45). Similarly, spontaneous NGAF transients within individual, isolated cochlear epithelial cells (i.e., not part of a propagated wave) had an average duration of 9.5 s (±3.4 SD; 14 isolated epithelial cells from 8 experimental trials in 4 explants) on the same time scale as rapid NADH dynamics in other tissues.

## 5 Discussion

*Ex vivo* imaging of semi-intact cochlear capsules using a genetically-encoded calcium sensor, GCaMP6f, enabled study of the tissue response to laser ablation of one to three outer hair cells. Serendipitously, the sensitive camera also revealed much weaker changes in fluorescence that did not rely on expression of the calcium sensor. This Non-GCaMP-Associated Fluorescence, NGAF, behaved similarly to damaged-evoked calcium waves studied previously in the cochlea and in skin. The NGAF waves propagated like those of previously published calcium waves, and like those, were diminished by purinoceptor blockade. Also, NGAF transients in isolated epithelial cells had a time course similar to the half-cycle duration of NADH oscillations found in other tissues (44, 45). As modeled for damage-evoked calcium waves in skin, these observations suggest that both intrinsic (intracellular oscillations of calcium, IP3 and NADH) and extrinsic (release of ATP through connexin hemi-channels) factors contribute to the damage-evoked signals in cochlear epithelia, as suggested previously (26).

Other studies of cochlear damage have identified additional molecular pathways that may be involved. Calcium homeostasis in small, electrically active hair cells is undoubtedly central to function and dysfunction (46, 47), including the generation of spontaneous calcium transients and synaptic maturation (48). Mitochondria serve as an important sink for cytoplasmic calcium, but additionally as a source of damaging reactive oxygen species during calcium overload (49, 50). It also has been shown that the damage-evoked calcium wave triggers activation of mitogen-activated kinases (ERK1/2) (51). These and stress-activated protein kinases (JNK and P38) have been implicated in the cochlear response to damage (52). Future studies can explore the impact of each of these pathways on the activation, speed and spread of epithelial calcium waves, and type II afferent activity.

The observations presented here provide a correlation, but do not establish causality. Are the type II afferents merely reporters of the epithelial signal, or do they contribute to its generation? The present data suggest that epithelial cells are the generators of the damage response. But in addition, as for somatic C-fibers, type II afferents could become ‘sensitized’ by prior trauma. Type II neurons express KCNQ potassium channels that contribute to the resting membrane conductance. ATP opens cation-selective P2X receptor channels, but also closes KCNQ channels via metabotropic P2Y receptors, further depolarizing the neuron (5). This increased input resistance of the type II afferent will make it more sensitive to glutamate release from OHCs, which, combined with the P2X-driven depolarization, could lower the threshold for making loud sound noxious. Presynaptically, ribbons of the OHCs increase in number and size after acoustic trauma (53), potentially providing stronger synaptic excitation to rouse previously silent type II contacts (27). In short, type II afferents may become better reporters of tissue damage after acoustic trauma. Whether this alters the probability, spread or persistence of epithelial waves remains to be determined.

Undoubtedly the pathogenesis of hyperacusis must involve still other processes.; especially considering that acoustically-evoked ear pain can continue for hours to days after sound exposure^2^ far beyond the minutes-long phenomena described to date. Such a prolonged time course suggests inflammation. Future research directions should include whether type II afferent signaling impacts immune cell activity in the cochlea. Type II afferents express neuropeptides such as CGRP (37, 38, 54), which in somatic C-fibers (55) modulates the activity of immune cells that proliferate after trauma (56). Thus, type II afferents could play a role in neuro-immune signaling as well. The cochlea provides another illustration of how neurons, epithelia and immune cells mediate the response to damage.

## Author contributions

MW: Conceptualization, Data curation, Formal analysis, Funding acquisition, Investigation, Methodology, Project administration, Visualization, Writing – review & editing. NN: Conceptualization, Data curation, Formal analysis, Investigation, Methodology, Visualization, Writing – review & editing. PF: Formal analysis, Funding acquisition, Project administration, Resources, Supervision, Writing – original draft, Writing – review & editing.

## Footnotes

^1^ https://hyperacusisresearch.org/

^2^ https://hyperacusisresearch.org/
